# Neurocognitive Performance Deficits Related to Immediate and Acute Blast Overpressure Exposure

**DOI:** 10.3389/fneur.2019.00949

**Published:** 2019-09-13

**Authors:** Christina R. LaValle, Walter S. Carr, Michael J. Egnoto, Anthony C. Misistia, Jonathan E. Salib, Alejandro N. Ramos, Gary H. Kamimori

**Affiliations:** Blast Induced Neurotrauma Branch, Center for Military Psychiatry and Neuroscience Research, Walter Reed Army Institute of Research, Silver Spring, MD, United States

**Keywords:** military, blast, cognition, breacher, practice effect

## Abstract

Addressing the concerns surrounding blast injury for the military community is a pressing matter. Specifically, sub-concussive blast effects, or those blast effects which do not yield a medical diagnosis but can result in symptom reporting and negative self-reported outcomes, are becoming increasingly important. This work evaluates explosive blast overpressure and impulse effects at the sub-concussive level on neurocognitive performance assessed with the Defense Automated Neurobehavioral Assessment (DANA) across seven breacher training courses conducted by the US Military. The results reported here come from 202 healthy, male military volunteer participants. Findings indicate that the neurocognitive task appearing most sensitive to identifying performance change is the DANA Procedural Reaction Time (PRT) subtask which may involve a sufficient level of challenge to reliably detect a small, transient cognitive impairment among a healthy undiagnosed population. The blast characteristic that was consistently associated with performance change was peak overpressure. Overall, this study provides evidence that increasing blast overpressure, defined as peak overpressure experienced in a training day, can lead to transient degradations in neurocognitive performance as seen on the DANA PRT subtask, which may generalize to other capabilities.

## Introduction

Blast injury is an increasing concern of Coalition Forces due to improvised explosives used during conflicts in Iraq and Afghanistan (1). Medical resources from both the Department of Defense and from the Department of Veterans Affairs have been allocated to address these injuries through prevention, recovery, and research. There are five blast injury mechanisms; this work concentrates on the primary mechanism, direct exposure to blast overpressure. Further, the focus here is overpressure exposure at a sub-concussive level, rather than a level of exposure that directly results in diagnosable injury from a single blast event. Accumulating evidence suggests that blast exposures can result in negative effects on the brain in absence of a medically diagnosable injury (2–5). The effects of these sub-concussive blast exposures are gaining attention in research and military communities concentrating on repeated exposures, deteriorated performance, and long-term health consequences. Importantly, studies finding deteriorated performance from sub-concussive overpressure exposure are not limited to preclinical models; negative effects are observed in naturalistic observation studies with human subjects [e.g., (4–7)]. Following overpressure exposures, participants in these studies reported elevated negative symptomology and exhibited declines in neurocognitive performance. Assessing sub-concussive blast effects can be difficult as they are hypothesized to be subtle and transient, especially among healthy, undiagnosed populations (8). The nature of low to moderate level blast exposure, or repeated exposures, inherently captures individuals with no detriment to performance or health, as well as those with some mild deteriorations.

Previous investigations on overpressure note military personnel reporting a symptom complex referred to as “breachers brain” which is characterized by issues with sleep disturbance, cognitive impairment, and headaches (5, 7, 9–12). “Breachers” are military and law enforcement personnel who use explosives in close proximity when gaining entry to a building by creating a “breach,” or opening in the building which allows them to interdict on some threat. The effects of “breachers brain” are not universal in the literature, and some research efforts have not revealed reliable evidence for negative consequences of blast [(8), for review]. Quantifying blast characteristics from low to moderate levels during operational training and accounting for non-blast factors that affect cognitive performance is complex. Open questions exist as to which blast components can be reliably measured in real time during operations and are associated with negative consequences. Additional elements include sufficient study duration, ample sample size, and consideration of appropriate covariate factors such as years of military service (4) and sleep (13) when assessing the effect of blast characteristics on neurocognitive performance.

To address aforementioned limitations, this study evaluated neurocognitive performance from 202 research volunteers who participated in a breacher training course involving heavy wall breaching charges where individual blast exposures were measured. Neurocognitive assessments were conducted immediately after breaching charges (<5 min) (Immediate effects) and at the end of the breacher training day (Acute effects): these are two pertinent time points relevant to combat effectiveness and health status. Both Tate et al. (5) and Carr et al. (4) demonstrated cognitive decrements at the end of day following breacher training lending to a hypothesized neurocognitive deficient in acute performance as measured in this study. Performance immediately following blast exposure is novel and presents insight for potential implications involved with going into a threat environment where speed of decision making impacts survivability. Immediate and acute performance were hypothesized to be negatively affected by blast exposure in neurocognitive tasks. Given that blast exposure levels experienced in regular military training on breaching, where blast-related medical injuries are not expected, we hypothesized that associated performance decrements would be small.

## Materials and Methods

All participants were provided a study briefing of research activities prior to granting written informed consent. The informed consent process and study activities were approved by the Walter Reed Army Institute of Research Institutional Review Board.

### Research Site and Volunteers

Seven data collections took place from August 2016 through July 2018 in conjunction with Urban Mobility Breachers Course (UMBC), a military breacher training course, conducted by the 35th Engineer Battalion at Ft. Leonard Wood, MO, USA. During training, students were exposed to as few as 2 and as many as 4 blast events from heavy breaching charges which included concrete wall charges [Net Explosive Weight (NEW): 10 lbs] and fence charges (NEW: 15 lbs). The UMBC was authorized to use 5 pounds per square inch (psi) as the safety level which was an authorized exception for this course.

The seven data collections comprised of 28–32 students, yielded a total sample of 202 volunteers. Students were male active duty military personnel, averaged 30 years of age (*SD* = 5.5, range 21–53 years) and fit for duty. The day before training, all subjects completed a demographics inventory with military history (e.g., duration of service), current health and health habits (e.g., hours of sleep per night), and self-reported information pertaining to medical history (e.g., history of head injury). Volunteers reported an average of 9.5 years of military service (*SD* = 4.9, range 2–25 years) and an average of 5.9 h sleep per night (*SD* = 1.2, range 3–9 h). Of the 202 participants, 66 of the volunteers reported a head injury during their military service.

### Blast Characterization

Blast exposure data were collected using Black Box Biometrics (B3: Rochester, NY) Blast Gauge sensors (generation 6). Three sensors were positioned on each volunteer to measure individual overpressure exposure: lower lateral deltoid region of the left and right shoulders, and helmet top. The left shoulder sensor data were used in analyses. In standard breacher training, personnel are positioned such that the sensor on their left shoulder has an approximately perpendicular orientation relative to the blast origin, yielding the closest measurement of incident overpressure (14). When sensors failed to trigger or when data was not recorded by the designated sensor and values were available for surrounding sensors, multiple imputation with regression-based replacement values were used. Imputations were conducted until convergence was achieved (generally 10–12 imputation cycles). Less than 10% of the total data set was imputed, with no changes to mean, standard deviation, or range (data dispersion was preserved). Prior to imputation, a variety of assessments were conducted to ensure missing data were distributed at random.

### DANA Administration

Neurocognitive performance was assessed using DANA Rapid (15). The DANA is a Java-based mobile application that runs on an Android operating system; it is portable and suitable for research in rugged field conditions (16). DANA Rapid is a 5-min task consisting of 3 subtasks, each of which uses visual stimuli and participants are to respond with a stylus to each trial as quickly as possible. These tasks are relatively simple, require minimal practice to asymptotic performance, and vary in cognitive complexity. For Simple Reaction Time (SRT), the participant taps a bullseye target when it is displayed (40 trials). For Procedural Reaction Time (PRT), a single digit is displayed from a pre-defined set of digits (2, 3, 4, or 5) and the participant taps either a left side target (for a “low” digit, 2 or 3) or a right target (for a “high” digit, 4 or 5) (32 trials). For Go/No-Go (GNG), a single figure of green or white color (“friend” or “foe,” respectively) is displayed in any of 6 windows of a building façade, and the participant taps a “fire” target when “foe” (white figure) is displayed (30 trials). The DANA was administered immediately (within 5 min; Immediate) after back-to-back explosive breaching charges and at end of day (25 min to 2 h after last breaching charge; Acute).

### Data Management and Analysis

The primary output measure for analysis was median reaction time (medRT) from SRT, PRT, and GNG subtasks. Outliers were assessed using interquartile range (IQR); trials with baseline medRT >3 IQR were removed from analyses. This was conducted for each subject and each subtest, removing 6 trials for SRT, 3 for PRT, and 3 GNG series from the analyses, approximately 2% of the DANA data. Performance change scores were calculated for DANA administered immediately after back-to-back breaching charges (Immediate effects) and at the end of the training day (Acute effects). The change scores were calculated by performance—baseline; thus, a negative change score indicates faster post-blast performance compared to baseline and a positive change score indicates slower post-blast performance compared to baseline.

Three independent variables were created from open-ended responses in the demographics inventory: duration of military service (Service; measured in years), quantity of sleep per night (Sleep; measured in hours; when a range was reported, the lower end of the range was used); and history of head injury prior to enrollment in the study that occurred during military service (Head Injury; yes or no). History of head injury was cross coded by two researchers to determine if head injuries described were incurred in the military (*r* = 0.87). Of the 4 total disagreements in the dataset, two were coding error and the remaining two were based on vocabulary. All disagreements were resolved via a second round of coding and discussion. The rationale for the criterion of head injury during military service and not using head injury prior to military service was to capture history of head injury as an adult (rather than as a child). The most common head injury found in the sample was improvised explosive device exposure.

Two blast characteristics were considered in analyses: Peak Overpressure, maximum recorded overpressure (psi) and Cumulative Impulse, overpressure recordings across the duration of the blast event (psi^*^ms). Blast characteristics were calculated for each individual using sensor readings from charges detonated immediately before taking the DANA (Immediate effects) and using sensor data from all breaching charges the individual was exposed to during the training day (Acute effects). The two blast variables were also dichotomized and analyzed to assess a “High” exposure group compared to a “Low” exposure group. “High” Peak Overpressure >5 psi (n_high_ = 73 and n_low_ = 129 for Immediate, and n_high_ = 83, and n_low_ = 119 for Acute); 5 psi was the level of overpressure for risk determined in a review of literature regarding blast, with risk based on rupture of the unprotected human eardrum (17). That review is the source of the exposure limit in use at military training sites. “High” Cumulative Impulse >25 psi (n_high_ = 55 and n_low_ = 147 for Immediate and n_high_ = 109, and n_low_ = 93 for Acute); 25 psi had been suggested in separate personal communications with subject matter experts.

Mixed-effects model analyses were used to compare Immediate and Acute DANA performance changes (Time) with blast characteristic (Peak Overpressure or Cumulative Impulse), Service, and Sleep as fixed effects and participant as a random effect to account for repeated (correlated) measures. Immediate and Acute performance were each evaluated using multiple regression to quantify the relationship between performance change and blast characteristics while accounting for Service and Sleep. Baseline DANA performance was included as a covariate in mixed-effects and multiple regression models involving DANA performance change to obtain more precise estimates (18). Sensitivity analyses were performed without baseline as a covariate and using models iteratively with the following variables: history of head injury; blast characteristics; Service; Sleep; history of head injury; and time (minutes) between last blast exposure and DANA administration. Statistical significance was set at *p* < 0.05 for all analyses. All data analyses were generated using SAS software, Version 9.3. Copyright © 2002–2011 SAS Institute Inc.

## Results

There was some variability among blast exposures in each of the seven data collections due to slight alterations within each training course. All participants experienced concrete wall breaching charges and some also experienced fence breaching charges (Table 1). Peak overpressures measured ranged from 2.57 psi to 9.17 psi, with an average peak overpressure of 4.61 psi (*SD* = 2.07). Also seen in Table 1 is that the number of charges decreased over the 2-year period of observation.

**Table 1:** Characterization of blast by training course.

**Data collection**	**Number of charges**	**Types of charges**	**Peak overpressure**	**Cum. Impulse**
1	3-4	CFCF	*Mean* = 4.35 (*SD* = 1.24) Min = 0.64, Max = 7.36	*Mean* = 32.59 (*SD* = 4.02) Min = 21.63, Max = 41.63
2	2-4	CFCF	*Mean* = 4.11 (*SD* = 1.10) Min = 1.39, Max = 6.81	*Mean* = 32.22 (*SD* = 7.63) Min = 18.60, Max = 45.82
3	2-4	CFCF	*Mean* = 4.18 (*SD* = 0.80) Min = 2.76, Max = 6.74	*Mean* = 33.93 (*SD* = 10.13) Min = 16.90, Max = 47.20
4	2	CC	*Mean* = 4.33 (*SD* = 0.62) Min = 2.90, Max = 6.02	*Mean* = 23.46 (*SD* = 2.22) Min = 19.50, Max = 28.80
5	2	CC	*Mean* = 4.55 (*SD* = 0.82) Min = 3.26, Max = 7.36	*Mean* = 26.11 (*SD* = 1.92) Min = 21.90, Max = 30.40
6	2	CC	*Mean* = 4.47 (*SD* = 1.14) Min = 2.57, Max = 7.80	*Mean* = 21.91 (*SD* = 3.71) Min = 15.05, Max = 30.36
7	2	CC	*Mean* = 4.38 (*SD* = 1.06) Min = 3.19, Max = 9.17	*Mean* = 23.98 (*SD* = 2.08) Min = 19.80, Max = 29.40

*Charge number varied within data collection based on team. Some teams could not receive as many charge opportunities as others due to number of students per group, or time commitment. CFCF indicates 2 concrete, and 2 fence charges, whereas CC indicates participants only had exposure to 2 concrete charges. Once charge frequency was reduced, cumulative impulse decreased over the courses evaluated*.

**Figure 1:**
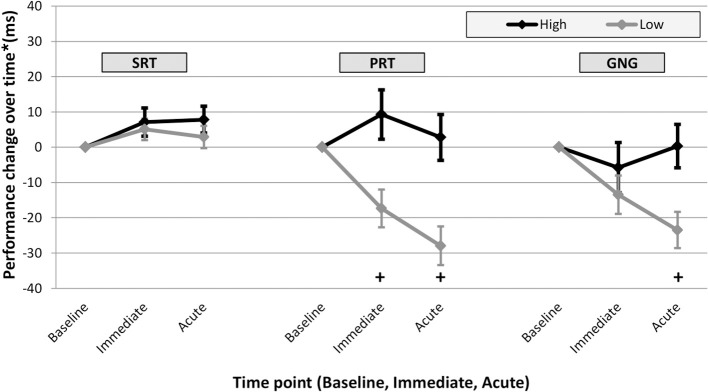
Adjusted* change score means (and standard error bars) for Immediate and Acute performance by DANA subtest. Change score are plotted over time for participants exposed to >5 psi peak overpressure (“High,” black), and participants exposed to 5 psi or less peak overpressure (“Low,” gray). In both PRT and GNG, the “High” exposure group mean performance change was statistically different from the “Low” group; participants with higher peak overpressure exposure have a reduced level of improvement. Further analyses (regression) resulted in statistically significant differences between “High” and “Low” groups in PRT at the Immediate and Acute time point in PRT and at the Acute time point in GNG as indicated below by “**+**.” ^*^Mean values estimated from regression using “High” and “Low” peak overpressure exposure groups.

Mixed-effects analyses resulted in statistically significant fixed effects in PRT for Time (Immediate vs. Acute) (Est. = 9.1, 95% CI [0.5, 17.8]), Peak Overpressure (Est. = 11.8, 95% CI [4.5, 19.2]), Service (Est. = 1.6, 95% CI [0.1, 3.2]), and Sleep (Est. = −7.5, 95% CI [-13.6, −1.3]). The fixed effects estimates suggested greater peak overpressure exposure (psi), less sleep hours, and more military service years were associated with less PRT performance improvement. There were no significant effects in SRT and GNG. Mixed-effects analyses using “High” and “Low” Peak Overpressure exposure groups resulted in statistically significant effects for Time (Est. = 9.3, 95% CI [0.7, 17.9]), “High” v “Low” Peak Overpressure group (Est. = 29.5, 95% CI [15.3, 43.6]), Service (Est. = 1.8, 95% CI [0.3, 3.4]), and Sleep (Est. = −7.9, 95% CI [-14.0, −1.8]) in PRT. Service was statistically significant in SRT (Est. = 0.9, 95% CI [0.0, 1.8]). “High” v “Low” Peak Overpressure group was statistically significant in GNG (Est. = 16.3, 95% CI [2.3, 30.4]). In PRT and GNG, the “High” exposure group had slower mean response times, equating to dampened performance improvement, compared to the “Low” group (Figure 1). The mixed-effects analyses indicated distinct evidence for an association between peak overpressure and performance change in PRT; while Acute performance (measured at the end of day) was significantly faster than Immediate performance (<5 min after breaching), there was less PRT performance improvement with greater peak overpressure exposure, more military service years, and less sleep duration. Moreover, personnel with peak overpressure exposure >5 psi showed less reaction time improvement compared to personnel with ≤5 psi peak overpressure exposure in the more complex neurocognitive subtasks.

**Figure 2:**
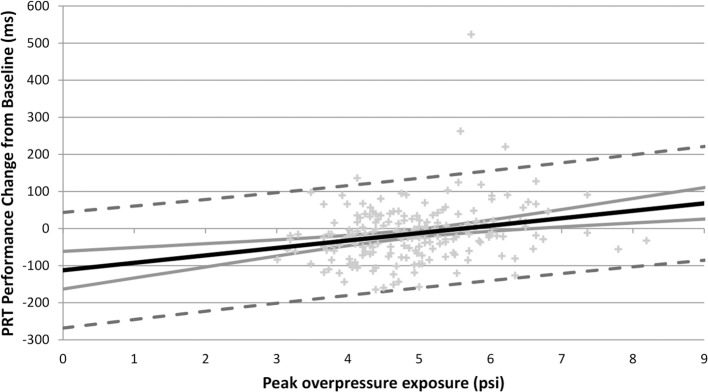
Peak overpressure exposure (psi) and acute PRT performance change (ms) scatter plot with regression line (black line), confidence interval (gray line), and prediction interval (dotted gray lines). Regression line illustrates association between higher peak overpressure and larger acute PRT performance change scores (indicating slower performance at the end of the training day compared to baseline). The confidence interval marks the boundary which contains the true best fit line with 95% probability. The predication interval shows the range of predicted performance values with corresponding peak overpressure exposure levels with 95% probability. Immediate PRT performance (not pictured) shows a similar trend (i.e., higher peak overpressure associated with larger Immediate PRT performance change scores), but with slightly less gradient.

Immediate and Acute performance changes and covariate variables were each examined to quantify the effect of blast exposure on neurocognitive performance with multiple regression. In PRT Immediate and Acute performance changes, increasing peak overpressure was associated with less improvement in performance compared to baseline (Figure 2 and Table 2). In analyses with dichotomized blast characteristics, personnel with peak overpressure >5 psi (“High”) had less improvement in performance than personnel with ≤5 psi peak overpressure exposure (“Low”) in Immediate and Acute PRT and Acute GNG (Figure 1 and Table 2). In examining both a continuous measure of blast exposure and categorizing exposure as “High” or “Low,” higher peak overpressure was consistently associated with slower performance compared to baseline in the PRT subtask of the DANA measured immediately after blast exposure (Immediate) and at the end of the training day (Acute).

**Table 2:** Multiple regression estimates (95% CI) for models assessing blast measures (continuous and dichotomized) and Immediate and Acute performance change by DANA subtests.

		**SRT**	**PRT**	**GNG**
**CONTINUOUS BLAST MEASURES**				
Immediate	Sleep	1.02 (−3.09, 5.12)	−6.38 (−13.62, 0.87)	−2.04 (−9.25, 5.17)
	Service	1.06 (0.00, 2.13)	**1.99 (0.13, 3.84)**	1.62 (−0.22, 3.47)
	Peak OP	−1.39 (−6.31, 3.52)	**9.17 (0.39, 17.94)**	0.23 (−8.50, 8.95)
	Sleep	0.97 (−3.11, 5.04)	−5.74 (−13.04, 1.55)	−1.78 (−8.94, 5.37)
	Service	1.00 (−0.04, 2.04)	**2.38 (0.55, 4.21)**	1.68 (−0.13, 3.49)
	Cumulative Imp	0.00 (0.00, 0.00)	0.00 (0.00, 0.00)	0.00 (0.00, 0.00)
Acute	Sleep	1.38 (−2.8, 5.55)	**−8.53 (−15.68**, **−1.39)**	−3.76 (−10.5, 2.98)
	Service	0.70 (−0.36, 1.76)	1.41 (−0.40, 3.21)	1.2 (−0.50, 2.9)
	Peak OP	2.65 (−2.29, 7.59)	**13.61 (4.97, 22.26)**	5.17 (−2.93, 13.27)
	Sleep	1.39 (−2.75, 5.52)	**−8.11 (−15.41**, **−0.82)**	−3.62 (−10.38, 3.14)
	Service	0.76 (−0.27, 1.79)	**1.94 (0.14, 3.75)**	1.39 (−0.29, 3.06)
	Cumulative Imp	0.64 (−0.02, 1.31)	0.76 (−0.44, 1.97)	0.33 (−0.77, 1.43)
**DICHOTOMOUS BLAST MEASURES**				
Immediate	Sleep	1.46 (−2.72, 5.64)	**−8.78 (−16.01**, **−1.55)**	−4.18 (−10.88, 2.52)
	Service	0.79 (−0.25, 1.83)	**1.82 (0.03, 3.61)**	1.28 (−0.38, 2.94)
	Peak OP H v L	3.37 (−6.63, 13.38)	**21.8 (4.31, 39.29)**	**18.28 (1.95, 34.62)**
	Sleep	1.52 (−2.65, 5.68)	**−8.05 (−15.41**, **−0.69)**	−2.83 (−9.57, 3.91)
	Service	0.81 (−0.23, 1.85)	**2.01 (0.20, 3.82)**	1.43 (−0.24, 3.09)
	Cumulative Imp H v L	−4.48 (−15.23, 6.27)	1.39 (−17.73, 20.51)	**17.98 (0.27, 35.69)**
Acute	Sleep	1.35 (−2.83, 5.54)	**−9.11 (−16.21**, **−2.00)**	−4.47 (−11.11, 2.17)
	Service	0.76 (−0.28, 1.81)	1.62 (−0.14, 3.39)	1.16 (−0.49, 2.81)
	Peak OP H v L	4.94 (−4.86, 14.75)	**30.71 (13.79, 47.63)**	**23.78 (7.84, 39.73)**
	Sleep	1.63 (−2.54, 5.80)	**−7.9 (−15.21**, **−0.58)**	−3.32 (−10.05, 3.42)
	Service	0.83 (−0.21, 1.87)	**1.96 (0.15, 3.77)**	1.34 (−0.33, 3.01)
	Cumulative Imp H v L	−2.14 (−11.78, 7.51)	8.9 (−8.37, 26.17)	12.83 (−3.45, 29.11)

Bold indicates statistically significant effect at p <0.05. OP, overpressure; Imp, impulse; Peak OP H v L, High group >5 psi overpressure compared to Low group ≤5 psi; Cumulative Imp H v L, High group >25 ms^*^psi cumulative impulse compared to Low group ≤25 ms^*^psi.

A variety of sensitivity analyses were conducted to test for robustness and consider the effects of other confounding factors. First, Head Injury was included in the mixed-effects and regression analyses. Head Injury was not significant in any model and did not change the significance of PRT results. Mixed-effects and regression analyses were conducted without the baseline performance covariate. For PRT, statistical significance of Time and blast characteristics remained (same as with baseline); however, Sleep and Service variables were not significant. For regression models, only Peak Overpressure remained statistically significant in PRT models. Peak Overpressure was significant in the GNG Acute effects model. Comparison regression analyses were conducted without Sleep and Service (baseline and blast characteristics only) and resulted in significant Peak Overpressure in Immediate and Acute PRT and GNG. Finally, the time (in minutes) between last blast exposure and taking the DANA was included in regression models; this variable was not significant and did not have an impact on results in terms of changes in significance of independent variables. These results provide an exhaustive perspective on factors to consider for analyses (i.e., baseline, Service, Sleep) and factors that do not impact results from these behavioral performance assessments (i.e., Head Injury, time between last blast, and test administration). The sensitivity analyses demonstrated that rigorous model fitting yielded more precise blast related performance estimates; for example, Acute PRT change resulted in smaller standard error (SE) for Peak Overpressure, from *SE* = 4.8 (with parameter estimate = 16.8 ms) in the Head Injury and no baseline model to *SE* = 4.4 (parameter estimate = 13.6 ms) in the no Head Injury with baseline model. Most importantly, sensitivity analyses demonstrated consistency and robustness in the relationship between greater peak overpressure exposure and less performance improvement in PRT.

## Discussion

The research reported here gives insight on warfighter readiness and informs decision-making in the use of force and tactics surrounding personnel working in kinetic environments. Results here showed that overpressure exposure can negatively affect neurocognitive performance. Those neurocognitive decrements can have negative consequences to mission success if reaction time, and potentially decision making, can be hampered by excessive overpressure exposure. This effect was clearest in the association between peak overpressure and PRT change from baseline. Specifically, greater peak overpressure was associated with reduced improvement in performance compared to baseline. Furthermore, as cumulative effects amass during long training or high operation days, the potentiality for errors in high stakes situations increases. This has impact on how decision-makers should consider sequencing events and cycling through personnel when tempo allows. At a minimum, the information here allows persons in charge to better assess risks against mission success and determine the best courses of action.

It must be noted that repeated performance on tasks like DANA is expected to have a practice effect, with performance improving across the first few administrations (19, 20). This finding, a reduced level of expected improvement when exposed to higher levels of blast overpressure, is consistent with other findings in comparable populations, most notably a reduction in practice effects among concussion cases in the U.S. Military Academy boxing program (21).

A potential means to account for the apparent sensitivity of the PRT subtest to performance decrement from blast exposure, relative to the other 2 subtests, SRT and GNG, may be found in in the additional burden the PRT task places on working memory and maintenance of cognitive sets. SRT and GNG require a single motor response to a visual target. GNG performance involves neurocognitive elements beyond SRT: spatial location of the target, 2 different colors of the target, and responding or withholding response based on the 2 different colors. PRT also involves further neurocognitive elements: 4 different targets, 2 sets within the 4 different targets (i.e., “low” vs. “high”), and 2 different motor responses (i.e., left vs. right). In other data from blast exposure, tasks involving spatial processing and response inhibition have not been found to be sensitive to performance deficits following low level blast exposure (4), whereas tasks with memory demand and maintenance of cognitive sets have shown to be sensitive (11, 22).

While performance changes associated with overpressure blast characteristics are measurable and statistically reliable, the effect is small, which is consistent with findings in other literature cited here. Building upon those findings already reported, this dataset is unique, yielding a much larger sample size of behavioral assessment data, critical for detecting small, subtle effects. These results warrant additional examination of what, if any, subtle slowing in neurocognitive reaction times have on mission success and long term health outcomes, especially in circumstances with larger exposure levels and/or greater complexity in neurocognitive challenge to performance, which could be expected to magnify these small effects observed in controlled training settings.

The blast characteristic that was consistently associated with performance change was peak overpressure. The effect of cumulative impulse was not consistent and did not affect performance change to the same extent as overpressure characteristics. Continuous and dichotomous blast measures were considered and produced similar results in PRT. Peak overpressure is the measure of blast that is typically reported in studies of this type. The evidence here is support for that convention. However, it is not known if peak overpressure is the sole driver for effects like performance impairment. It may be the case that the acoustic insult that also grows larger with larger peak overpressure is a contributing, or even primary, mechanism of effect (23, 29).

Sleep and duration of service appear to have an impact on neurocognitive performance in the immediate and acute results. It is well-documented that sleep affects neurocognitive performance (24) and this association has been observed in preliminary analyses of data like those reported here (13, 25); this work used a simple self-report of sleep that sufficiently accounted for variability in analyses. Duration of service and occupational exposure to blast have also been demonstrated to affect cognition (7). The results of this study emphasize the importance of considering additional factors that affect performance when assessing how blast characteristics affect performance as demonstrated in the sensitivity analyses with GNG where peak overpressure was significant when Service and Sleep were removed from the model. In addition, if covariates that are known to affect performance are excluded from blast-performance analyses, it is critical to demonstrate the sample is homogenous among mediating demographic factors. While history of prior head injury incurred in the military, most often from blast, may impact neurocognitive performance, the information used to evaluate this effect was tenuous, at best, and needs additional scrutiny such in an assessment that is more judicious than self-report.

These data serve as reference for an exposure threshold that could be used as a marker for potential performance decrement in individual Soldiers. While medical imaging and a variety of approaches to biomarker assessments have made and continue to make significant advances, behavioral assessments, like the DANA reported here, remain the coin of the realm in evaluating functional impairments in field settings. Importantly, cumulative impulse exposure in excess of 25 psi^*^ms, and overpressure in excess of 5 psi seem to relate to detrimental performance reliably. The interaction between these two numbers is a critical point of future investigation. Knowing how the composition of overpressure and cumulative impulse influence each other with regards to detriment will be necessary for blast program monitoring success. Exposures that lead to detrimental performance (overpressure >5 psi, Impulse >25 psi^*^ms) are encountered in current training evaluated here, and suggest that current minimum safe distance (MSD) calculations are rife for improvement when applied to complex environments. However, this is not to say that all possible mechanisms to accumulate 25 psi^*^ms exposure in a day are equally detrimental.

Notably in the conduct of observational studies like this of routine military training, this work catalogs the evolution of breacher military training over a 2-year period. The number of charges can be seen to decrease as units organically refine training to maximize preparedness of warfighters while being mindful of resource and health considerations. This work focused on neurocognitive performance as assessed via the DANA. The speculation reported here, on the sensitivity advantage that PRT may connote, can be specifically tested in new data collection. Also, new testing may include clinical assessments like the California Verbal Learning Test, Trail Making Test, the Wechsler Test of Adult Reading, among others, as well-established measures that can add to clinical neuropsychological insight. There is also evidence to suggest additional blast assessment, like high-speed footage to help detect and assess particle velocity, may be worthwhile (26). Field collections do not always lend themselves to such environments where particle velocity measurements can be made, but research suggests that where possible, this facet should be considered (27, 28). Insight gained by targeted expansion of neurocognitive assessment strategies to reveal the mechanisms of deficit that are occurring in service members, as well as to follow recovery from those detriments would advance the understanding of blast effects.

In sum, this work provides evidence not only for the maintenance of Soldier health long-term, but for the safety of personnel in harms way. Knowing that neurocognitive performance related to decision making and reaction time can be compromised by blast exceeding certain thresholds for peak and cumulative pressure provides pertinent information for decision making. Knowing when to switch in personnel for “fresh legs” during operation to prevent severe injury or death is not just an ethical obligation of our forces, but a significant advantage in maintaining force readiness in austere environments.

## Data Availability

The datasets for this study will not be made publicly available because Data used in the preparation of this article reside in the Department of Defense (DOD) and National Institutes of Health (NIH)-supported Federal Interagency Traumatic Brain Injury Research Informatics Systems (FITBIR) in [FITBIR-STUDY0000353]. This manuscript reflects the views of the authors and does not reflect the opinions or views of the DOD or the NIH.

## Ethics Statement

This study was carried out in accordance with the recommendations of the Walter Reed Army Institute of Research (WRAIR) Human Subjects Protection Branch (HSPB) and Information Review Board (IRB) with written, informed consent from all participants. All subjects gave written informed consent in accordance with the declaration of Helsinki. The protocol was approved by the WRAIR IRB.

## Author Contributions

CL wrote manuscript, analyzed data, managed protocol and human subject regulatory files. WC wrote, revised, and oversaw the creation of the manuscript. ME assisted in writing the manuscript. AM, JS, and AR assisted in the data collection. GK developed the research agenda and oversaw all aspects of the research.

### Conflict of Interest Statement

The authors declare that the research was conducted in the absence of any commercial or financial relationships that could be construed as a potential conflict of interest.
